# HLA molecular mismatches and induced donor-specific tolerance in combined living donor kidney and hematopoietic stem cell transplantation

**DOI:** 10.3389/fimmu.2024.1377535

**Published:** 2024-03-27

**Authors:** Aleksandar Senev, Anat R. Tambur, Vasilis Kosmoliaptsis, Hannah Charlotte Copley, Cynthia García-Sánchez, Crystal Usenko, Suzanne T. Ildstad, Joseph R. Leventhal

**Affiliations:** ^1^ The Comprehensive Transplant Center (CTC) at Northwestern University Feinberg School of Medicine, Northwestern University, Chicago, IL, United States; ^2^ Department of Surgery, Addenbrooke’s Hospital, University of Cambridge and National Institute for Health Research Cambridge Biomedical Research Centre, Cambridge, United Kingdom; ^3^ Institute for Cellular Therapeutics, University of Louisville, Louisville, KY, United States

**Keywords:** HLA, kidney transplantation, stem cell transplantation, tolerance, HLA epitope analysis, HLA molecular mismatches

## Abstract

**Introduction:**

We investigated the potential role of HLA molecular mismatches (MM) in achieving stable chimerism, allowing for donor-specific tolerance in patients undergoing combined living donor kidney and hematopoietic stem cell transplantation (HSCT).

**Methods:**

All patients with available DNA samples (N=32) who participated in a phase 2 clinical trial (NCT00498160) where they received an HLA mismatched co-transplantation of living donor kidney and facilitating cell-enriched HSCT were included in this study. High-resolution HLA genotyping data were used to calculate HLA amino acid mismatches (AAMM), Eplet MM, three-dimensional electrostatic mismatch scores (EMS-3D), PIRCHE scores, HLA-DPB1 T-cell epitope group MM, HLA-B leader sequence MM, and KIR ligands MM between the donor and recipient in both directions. HLA MM were analyzed to test for correlation with the development of chimerism, graft vs. host disease (GvHD), *de novo* DSA, and graft rejection.

**Results:**

Follow-up time of this cohort was 6–13.5 years. Of the 32 patients, 26 developed high-level donor or mixed stable chimerism, followed by complete withdrawal of immunosuppression (IS) in 25 patients. The remaining six of the 32 patients had transient chimerism or no engraftment and were maintained on IS (On-IS). In host versus graft direction, a trend toward higher median number of HLA-DRB1 MM scores was seen in patients On-IS compared to patients with high-level donor/mixed chimerism, using any of the HLA MM modalities; however, initial statistical significance was observed only for the EMS-3D score (0.45 [IQR, 0.30–0.61] vs. 0.24 [IQR, 0.18–0.36], respectively; p=0.036), which was lost when applying the Bonferroni correction. No statistically significant differences between the two groups were observed for AAMM, EMS-3D, Eplet MM, and PIRCHE-II scores calculated in graft versus host direction. No associations were found between development of chimerism and GvHD and non-permissive HLA-DPB1 T-cell epitope group MM, HLA-B leader sequence, and KIR ligands MM.

**Conclusion:**

Our results suggest an association between HLA-DRB1 molecular mismatches and achieving stable chimerism, particularly when electrostatic quality of the mismatch is considered. The non-permissive HLA-DPB1 T-cell epitope group, HLA-B leader sequence, and KIR ligands MM do not predict chimerism and GvHD in this combined kidney/HSCT transplant patient cohort. Further work is needed to validate our findings.

**Clinical trial registration:**

https://clinicaltrials.gov/study/NCT00498160, identifier NCT00498160.

## Introduction

The genetic disparity between donor and recipient in the human leukocyte antigen (HLA) system plays a central role in allograft rejection after kidney transplantation. To avoid allograft loss due to rejection, patients need to be on lifelong immunosuppressive medication. While the availability of new potent immunosuppressive drugs has led to a dramatic decrease in the incidence of early acute rejection in kidney transplant recipients, patient mortality due to complications related to the chronic use of immunosuppression drugs remains a significant problem. For instance, there is considerable evidence showing that kidney transplant recipients have a two- to fourfold higher incidence of cancer than the general population (1). Therefore, attention is being focused on reducing the burden of immunosuppression to minimize long-term adverse effects.

Immune tolerance denotes a state in which the transplant recipient’s immune system accepts the donor organ while responding normally to foreign antigens and pathogens. Establishment of post-transplant immune tolerance might allow for the discontinuation of chronic immunosuppression to prevent transplant rejection. The spontaneous development of tolerance in kidney transplant recipients has been observed in small numbers of patients, associated with evidence of donor-specific immunomodulation (2). Alternatively, tolerance can be intentionally induced using therapeutic cell transfer with donor hematopoietic stem cells (HSCs) (3–5). Establishment of persistent donor hematopoietic stem cell chimerism has proven to be an effective strategy for tolerance induction in mismatched donor/recipient pairs (6). This hematopoietic chimerism-based tolerance is attributed in part to clonal deletion of alloreactive cells where exposure to donor antigen during reconstitution of the immune system deletes donor-reactive T cells and, along with other peripheral tolerance mechanisms, leads to long-term robust tolerance (7).

We have conducted a phase 2 clinical trial of combined living donor kidney and stem cell transplantation to induce transplant tolerance The protocol was based upon infusion of donor HSCs enriched for tolerogenic CD8+TCR− facilitating cells (FCR) and non-myeloablative conditioning (6). Facilitating cells are bone-marrow-derived populations of nucleated cells that help promote stem cell engraftment with a reduced incidence of GVHD. Facilitating cells were first described by Ildstad et al. as a bone marrow-derived CD8+TCR− cell population that enables engraftment of hematopoietic stem cells across HLA barriers (8). Stable chimerism was achieved in 26 subjects, two subjects did not engraft, and transient chimerism was observed in eight subjects (6). Patients with no donor stem cell engraftment and transient chimerism resumed endogenous hematopoiesis and were maintained on low-dose immunosuppression (IS); however, some of them developed allograft rejection.

A significant potential complication of chimerism-based approaches to tolerance is graft versus host disease (GvHD), where donor immune cells, mainly T cells, attack the recipient. Severe GvHD is a significant cause of morbidity and mortality in stem cell transplantation. Acute and chronic GvHD cases were reported in trials with HLA-matched transplant pairs; however, the risk is significantly higher in HLA-mismatched transplant pairs. In our phase 2 trial, we have observed two cases of severe acute GvHD (5, 6).

It remains unclear what the key determinants of clinical outcomes are with the FCR graft engineering approach to tolerance induction without causing GvHD. A more detailed evaluation of the HLA disparities between donor and recipient at the HLA molecular mismatch (epitope) level with the use of high-resolution HLA molecular typing methods has the potential for a more precise prediction of posttransplant alloimmune responses (9). Studies evaluating the HLA mismatches at the molecular level have shown greater clinical importance of HLA class II molecules compared to class I HLA molecules (10–12). Therefore, in this study, we hypothesized that a lower HLA molecular mismatch load for HLA class II molecules would be associated with the successful establishment of stable chimerism and achievement of induced tolerance in the absence of GvHD in HLA mismatch transplant recipients receiving a combined kidney and FCR transplant. Additionally, we investigated if non-permissive HLA-DPB1 T-cell epitope group mismatches, HLA-B leader sequence mismatch, and KIR ligands mismatches would predict the clinical outcome in this combined transplant setting.

## Materials and methods

### Study population

In 2009, our group initiated a phase 2 clinical trial (NCT00497926) to induce tolerance in mismatched related and unrelated recipients of living donor renal allografts using donor HSCT engineered to be enriched for FC (FCR) (6). A total of 42 subjects were enrolled, and 37 have been transplanted with ABO-compatible donor as part of this trial. A total of 36 of the 37 transplant patients who were part of this clinical trial at Northwestern University and received a kidney transplant and hematopoietic stem/facilitating cell induction protocol from ABO-compatible and HLA-mismatched donors were considered for this study. All clinical data were collected prospectively for the purpose of the clinical trial and is available for the current study analyses. All protocols were approved by the Northwestern University (NWU) Food and Drug Administration (IDE 13947). Informed consent was obtained for all donors and recipients.

### Hematopoietic stem cells and kidney conditioning regimen

The algorithm for conditioning, kidney and FCR transplant, and maintenance immunosuppression was published previously (13). At least 2 weeks before the kidney transplant, donors were mobilized with granulocyte colony-stimulating factor, and apheresis was performed. The apheresis product was shipped, in a controlled-temperature container, to the Institute for Cellular Therapeutics at the University of Louisville, where it was processed under Food and Drug Administration approval to obtain the engineered donor stem cell transplantation product that contains the HSCs, FCs, and progenitor cells. This product, termed the FCR, was then cryopreserved and shipped to the transplant center for later infusion.

Recipients were conditioned non-myeloablatively with fludarabine (30 mg/m^2^ per dose, days −5, −4, and −3), cyclophosphamide (50 mg/kg per dose, day −3 and +3), and 200 centigray (cGy) total body irradiation (day −1). Two days before the transplant, recipients received tacrolimus and mycophenolate mofetil, which were continued as maintenance immunosuppression. The final FCR product was infused on the first day after the living donor kidney transplant. None of the recipients received antibody induction therapy.

### Chimerism testing

Chimerism was determined through the genotyping of simple sequence-length polymorphisms that encode short tandem repeats at Northwestern University or an independent laboratory (LabCorp, Burlington, NC). For lineage chimerism testing, B cells (CD19+), T cells (CD3+), and/or myeloid cells (CD66B+) were sorted from whole blood and then analyzed by molecular short tandem repeat typing. The assay has a sensitivity of approximately 2%–5%, and internal controls were performed for each assay to define the sensitivity. Tolerance was defined by the ability to wean patients off IS and maintain stable kidney function without biopsy-proven acute rejection. The institutional review boards approved the protocol as an investigational device exemption by the Food and Drug Administration.

### HLA typing, HLA mismatch calculations, and detection of circulating anti-HLA antibodies

Recipients and donors of this cohort with available DNA samples were genotyped retrospectively at high-resolution level for 11 HLA loci (HLA-A, HLA-B, HLA-C, HLA-DRB1, HLA-DRB345, HLA-DQA1, HLA-DQB1, HLA-DPA1, and HLA-DPB1) using the following methods: next-generation sequencing (Illumina), sequencing-based typing (Applied Biosystems), and sequence-specific oligonucleotides probes (high‐resolution XR LABType, Luminex).

The high-resolution HLA genotypes for all 11 loci were uploaded to HLAMatchmaker (ABC_v4; DRDQDP_v3.1), Cambridge HLA Immunogenicity algorithm (14, 15), and PIRCHE-II (v3.3.64) software to calculate the HLA molecular mismatches. We obtained the total HLA molecular mismatch scores: total and eplet mismatches, total amino acid mismatches, three-dimensional electrostatic mismatch score (EMS-3D) (16), and PIRCHE-II. The HLA molecular mismatch score load was calculated and defined for each transplant per locus and per class in a bi-directional way [host versus graft (HvG) and graft versus host (GvH) direction], and the association of these scores with the risk of developing stable chimerism, GvHD, *de novo* DSA, and kidney graft rejection was examined.

To determine the permissiveness and non-permissiveness of DPB1 mismatches, we employed the DPB1 T-Cell Epitope Algorithm v2.0, available at https://www.ebi.ac.uk/ipd/imgt/hla/matching/dpb_v2/. To analyze HLA-B types of the HLA-B leader sequences depending on methionine (M) or threonine (T) at position −21 and compare them for mismatches between the patient and the donor, we used the tool available on the IPD-IMGT/HLA website (https://www.ebi.ac.uk/ipd/imgt/hla/matching/b_leader/). Finally, to group KIR ligands into the three major categories based on the KIR-binding epitope in HLA-C and HLA-B and calculate the KIR ligand mismatches, we used the KIR Ligand Calculator, also available on the IPD-IMGT/HLA website. We did not genotype the recipients for KIR receptors.

Anti-HLA antibodies were systematically monitored at the Transplant Immunology Laboratory at Northwestern University. All sera were first screened using the FlowPRA Class I and II Screening Test (One Lambda) by flow cytometry. In case of positive screening, the donor specificity was assessed using single antigen bead (SAB) assays: LABScreen Single Antigen Class I and Class II kits (One Lambda) or LIFECODES^®^ Single Antigen Assays (LSA) class I and class II kits (Immucor. Inc). Monitoring for *de novo* DSA post-transplant was performed prospectively as the standard of care using the same approach of flow PRA screening and, if positive, Luminex SAB testing. Dilution was used as a measure to remove potential inhibition, and all 11 HLA loci were considered to define the presence of DSA.

### Kidney allograft biopsies

Protocol allograft kidney biopsies were performed at 6, 12, and 24 months as per clinical trial design, with further biopsies being done at the discretion of the investigator.

### Statistical analyses

Patient and donor characteristics are described by means and standard deviation (SD) or medians and interquartile range (IQR), the two-sample t-test for the continuous variables, and the Wilcoxon test for the comparison of medians. All p-values of 0.05 or less were considered to indicate statistical significance. For the comparisons with the individual HLA molecules, we applied the Bonferroni correction for multiple testing with a significance threshold of p < 0.008. We used SAS (v9.4; SAS Institute, Cary, NC) and GraphPad Prism software (v9.3; GraphPad Software, San Diego, CA) for the statistical analyses.

## Results

### Study population and demographic characteristics

Of the 36 HLA-mismatched transplant pairs who were part of the trial, 32 pairs had DNA samples available for high-resolution HLA typing and were included in this study. The median (IQR) follow-up time for these patients was 8 (6–8) years. **Table 1** shows the main demographic and immunological characteristics of the study cohort. The recipients’ mean ( ± SD) age was 42.5 ( ± 11.3) years. The majority of them were white (87.5%) men (78.1%) and received their first kidney transplant (93.8%). Similarly, the mean ( ± SD) age of the donors was 41.1 ( ± 10.4) years, and most of them were men (78.1%), with half of them (50%) being living-related donors.

**Table 1:** Main demographic, pretransplant clinical characteristics of the study population (n=32).

Cohort characteristics	Total (n=32)
*Recipient demographics*	
Sex (male), n (%)	25 (78.1)
Age at transplant (years), mean ± SD	42.5 ± 11.3
Previous transplantation, n (%)	30 (93.8)
Caucasian ethnicity, n (%)	28 (87.5)
*Donor demographics*	
Sex (male), n (%)	20 (62.5)
Age at transplant (years), mean ± SD	41.1 ± 10.4
Unrelated living donor, n (%)	16 (50.0)
*Transplant characteristics*	
Pretransplant HLA antibodies by flow PRA, n (%)	17 (53.1)
HLA class I antibodies by flow PRA, n (%)	14 (43.8)
HLA class II antibodies by flow PRA, n (%)	7 (21.8)
Total % of HLA antibodies by flow PRA, median (IQR)	2 (0-8)
Pretransplant donor-specific HLA antibodies, n (%)	0 (0)
*HLA mismatches in host vs. graft direction*	
HLA-ABCDRDQ antigen mismatches (0-10), mean ± SD	5.8 ± 2.5
A antigen, mean ± SD	1.1 ± 0.7
B antigen, mean ± SD	1.3 ± 0.7
C antigen, mean ± SD	1.3 ± 0.7
DR antigen, mean ± SD	1.7 ± 1.0
DQ antigen, mean ± SD	0.9 ± 0.6
Total HLA allele mismatches (0-18)*, mean ± SD	8.3 ± 3.2
Total AAMM*, median (IQR)	52.5 (34.8–68.5)
Total EMS-3D score*, median (IQR)	2.1 (1.1–2.4)
Total eplet mismatches*, median (IQR)	65.0 (42.0–76.5)
Total PIRCHE-I score*, median (IQR)	2.5 (0.0–7.3)
Total PIRCHE-II score*, median (IQR)	221.0 (130.0–343.0)
*HLA mismatches in graft vs. host direction*	
HLA-ABCDRDQ antigen mismatches (0–10), mean ± SD	5.8 ± 2.5
A antigen, mean ± SD	1.0 ± 0.8
B antigen, mean ± SD	1.3 ± 0.8
C antigen, mean ± SD	1.3 ± 0.8
DR antigen, mean ± SD	1.7 ± 0.9
DQ antigen, mean ± SD	0.9 ± 0.6
Total HLA allele mismatches (0-18)*, mean ± SD	8.0 ± 3.3
Total AAMM, median (IQR)	58.5 (39.3–68.0)
Total EMS-3D score, median (IQR)	1.8 (1.3–2.2)
Total eplet mismatches, median (IQR)	92.0 (70.5–117.0)
Total PIRCHE-I score, median (IQR)	8.0 (0.0–21.0)
Total PIRCHE-II score, median (IQR)	92 (53–161)

*Two transplant pairs did not have DPB1 typing.

Pretransplant HLA antibodies were present in 17 (53.1%) recipients, but none had HLA antibodies specific to the donor (DSA). The total mean ( ± SD) number of HLA antigen and allele mismatches in the HvG direction were 5.8 ± 2.5 and 8.3 ± 3.2, respectively. The highest mean ( ± SD) number of individual HLA antigen mismatches was found for HLA-DR (1.7 ± 1.0). In terms of different molecular mismatch modalities, the total (HLA class I and II) median (IQR) numbers were 52.5 (34.8–68.5) for amino acid mismatches, 65.0 (42.0–76.5) for eplet mismatches, 2.1 (1.1–2.4) for EMS-3D, 2.5 (0.0–7.3) for PIRCHE-I and 221.0 (130.0–343.0) for PIRCHE II score. Molecular mismatch scores were comparable for all different molecular mismatch modalities in the GvH direction (**Table 1**).

### Patient clinical outcomes and development of *de novo* HLA-DSA

**Figure 1:**
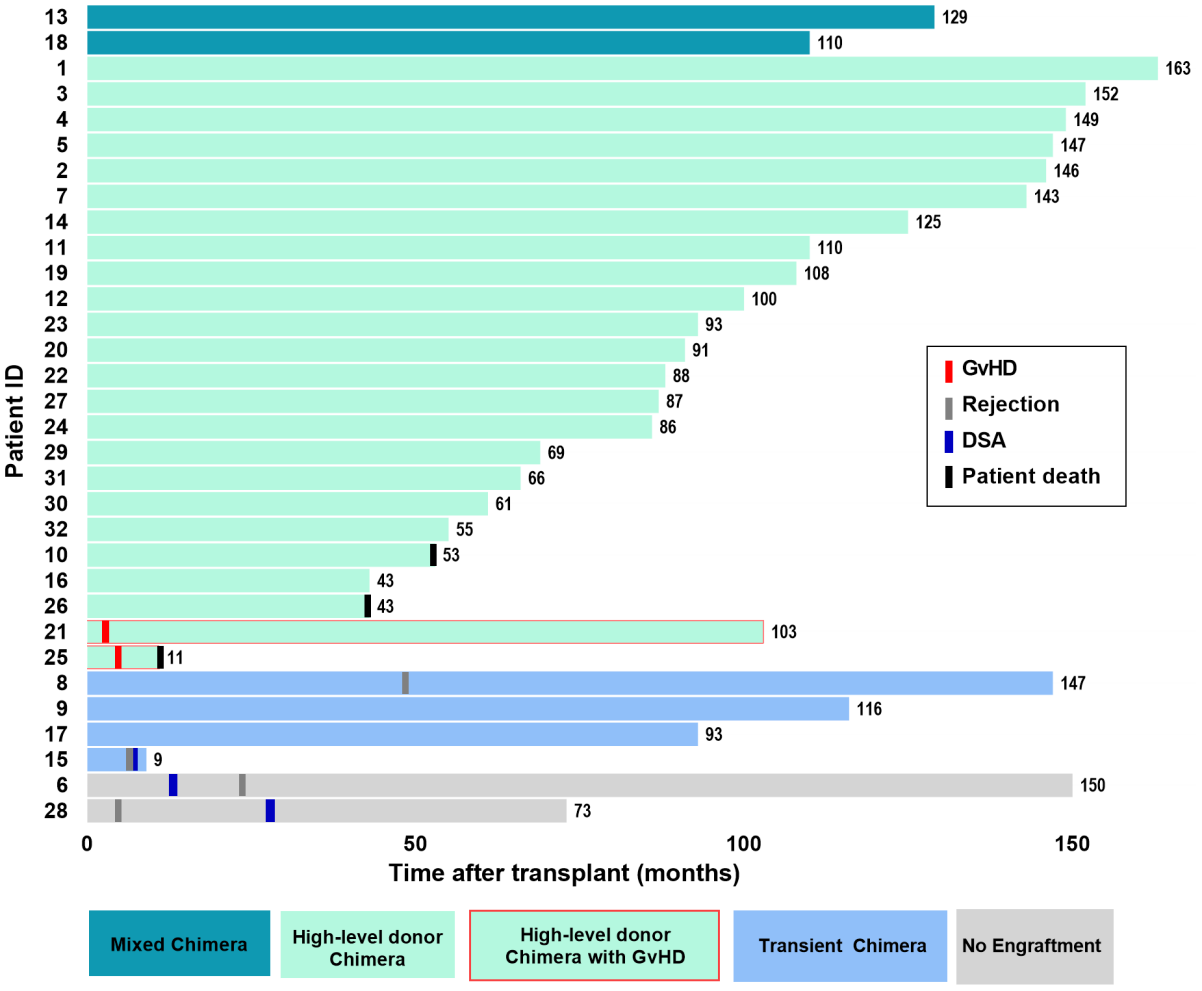
Graphical representation of individual post-transplant timeline and clinical outcome for 32 patients. Each bar represents one patient, and different colors represent the outcome of the stem cell transplant (light green—high-level donor chimerism; darker green—mixed chimerism; blue—transient chimerism and gray—no engraftment). The colored vertical line represents other clinical outcomes (red -GvHD, gray—TCMR/ABMR rejection; blue—*de novo* DSA; black—patient death).

All recipients were initially maintained on tacrolimus and mycophenolate-based IS. At 6 months, if chimerism, stable renal function, absence of HLA-DSA, and a normal protocol graft biopsy were noted, then mycophenolate was discontinued. Tacrolimus was weaned over the next 6 months and fully withdrawn at 1 year after the combined kidney and stem-cell transplant if the aforementioned 6-month endpoints were met. A total of 26 patients in our cohort achieved stable donor chimerism, enabling 25/26 to fully withdraw from immunosuppression; one subject with high-level donor stable chimerism developed severe GvHD and died 9 months posttransplant. Patients who had transient chimerism were able to resume endogenous hematopoiesis and were maintained on low-dose immunosuppression (On-IS), with most being converted to monotherapy. Two patients with high-level donor chimerism developed GvHD. T-cell-mediated rejection was observed in four subjects with transient/no donor chimerism (**Figure 1**).

**Figure 2:**
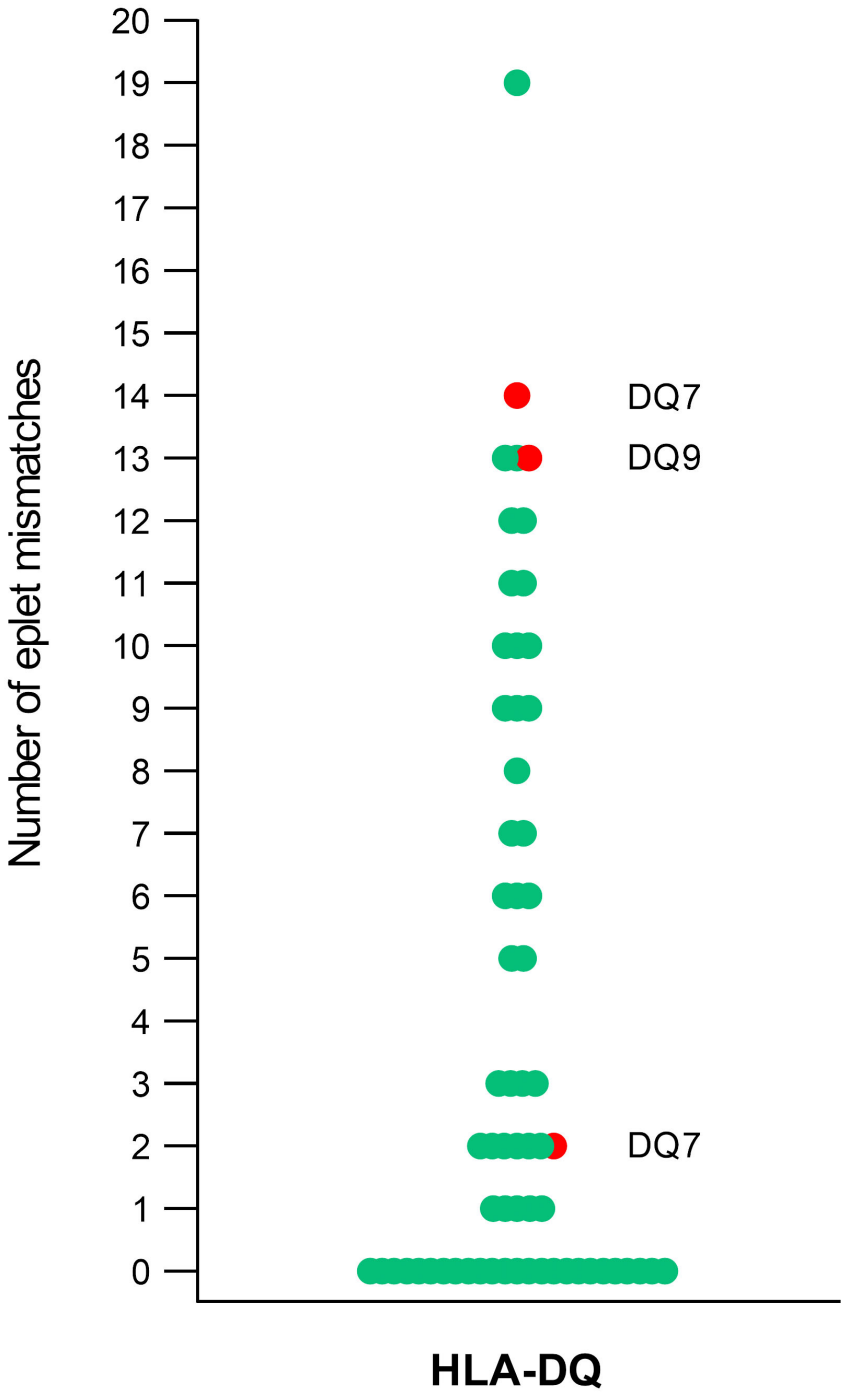
Distribution of HLA eplet mismatches for HLA-DQα_1_β_1_. Each data point (circle) represents HLA eplet mismatch score calculated per single HLA molecule in HvG directionality. In red are shown the three eplet mismatch scores with the corresponding HLA specificity that led to the development of HLA-DQα_1_β_1_ *dn*DSA.

During the follow-up, only three (9%) patients developed *de novo* (dn) DSA, most against DQ specificities. One patient with transient chimerism developed HLA class I dnDSAs, while the other two patients who had no engraftment developed HLA class II dnDSAs. One dnDSA was against DR (DR53), and three were against DQ (two DQ7 and one DQ9). **Figure 2** shows the distribution of HLA eplet mismatches for a single HLA-DQα1β1 molecule as the most common dnDSA. While two of the dnDSA had an eplet load >9 (11), the third DSA (DQ7) was developed across a mismatch of only two eplets.

### Differences in HLA mismatches between patients On-IS vs. patients with stable chimerism

We compared the AAMM, EMS-3D, EpMM, and PIRCHE-II scores between patients On-IS versus those with stable chimerism in the HvG direction (**Table 2**). We looked at both total scores and scores per HLA class and molecule. Although all different modalities showed a trend toward significance for the DRB1 molecule, only the EMS-3D score indicated statistical significance between patients On-IS vs. patients with stable chimerism (0.45 [0.30–0.61] vs. 0.24 [0.18–0.36], p=0.036), in which the significance was lost after applying the Bonferroni correction. In the GvH direction, no trend or statistically significant differences were observed between the two groups for all different molecular mismatch modalities (**Table 3**).

**Table 2:** Donor HLA molecular mismatches in host vs. graft direction.

HLA mismatches	Patients On-IS (N=6)	Patients Off-IS (N=26)	Wilcoxon exact test p-value
*HLA Amino Acid mismatches (AAMM)*			
Total AAMM, median (IQR)	66.5 (36.0–83.0)	52.0 (28.0–64.0)	0.26
AAMM for HLA class I, median (IQR)	24.0 (19.0–28.0)	25.0 (17.0–31.0)	0.99
AAMM for HLA-A, median (IQR)	7.5 (0.0–10.0)	9.0 (3.0–13.0)	0.50
AAMM for HLA-B, median (IQR)	11.0 (4.0–14.0)	7.5 (3.0–10.0)	0.28
AAMM for HLA-C, median (IQR)	7.0 (7.0–8.0)	7.5 (2.0–11.0)	0.79
AAMM for HLA class II, median (IQR)	41.5 (25–55)	26 (16–39)	0.15
AAMM for HLA-DRB1, median (IQR)	17.0 (13–29)	13 (6–17)	0.14
AAMM for HLA-DQA1/B1, median (IQR)	17 (11–42)	15 (7–22)	0.41
AAMM for HLA-DPA1/B1, median (IQR)	7 (2 –14)	2 (0–8)	0.27
*HLA EMS-3D score*			
Total EMS-3D score, median (IQR)	2.30 (1.11–2.99)	2.05 (0.96–2.24)	0.19
EMS-3D score for HLA class I, median (IQR)	1.44 (0.65–1.70)	1.20 (0.80-1.60)	0.65
EMS-3D score for HLA-A, median (IQR)	0.42 (0.0–0.53)	0.34 (0.23–0.52)	0.75
EMS-3D score for HLA-B, median (IQR)	0.45 (0.29–0.58)	0.33 (0.12–0.56)	0.46
EMS-3D score for HLA-C, median (IQR)	0.47 (0.30–0.70)	0.43 (0.29–0.66)	0.86
EMS-3D score for HLA class II, median (IQR)	0.91 (0.63–1.29)	0.63 (0.44–0.95)	0.13
EMS-3D score for HLA-DRB1, median (IQR)	0.45 (0.30–0.61)	0.24 (0.18–0.36)	0.036*
EMS-3D score for HLA-DQA1/B1, median (IQR)	0.38 (0.32–0.70)	0.41 (0.2–0.56)	0.49
EMS-3D score for HLA-DPA1/B1, median (IQR)	0.18 (0.1–0.34)	0.07 (0.0–0.24)	0.31
*Eplet mismatches*			
Total eplet mismatches, median (IQR)	64.0 (60.0–77.0)	65.0 (37.0–83.0)	0.38
HLA class I eplet mismatches, median (IQR)	28.0 (19.0–37.0)	32.0 (15.0–37.0)	0.80
HLA-A eplet mismatches, median (IQR)	13.0 (9.0–14.0)	12.0 (6.0–19.0)	0.86
HLA-B eplet mismatches, median (IQR)	11.0 (3.0–14.0)	10.0 (5.0–15.0)	0.97
HLA-C eplet mismatches, median (IQR)	7.5 (4.0–10.0)	11.0 (8.0–12.0)	0.26
HLA class II eplet mismatches, median (IQR)	40.0 (37.0–50.0)	33.5 (18.0–44.0)	0.18
HLA-DRB1 eplet mismatches, median (IQR)	16.0 (13.0–23.0)	13.0 (8.0–15.0)	0.08
HLA-DQA1/B1 eplet mismatches, median (IQR)	26.5 (20–30.0)	21.5 (11.0–30.0)	0.49
*PIRCHE-II score*			
Total PIRCHE-II score, median (IQR)	487.0 (436.0–617.0)	367.0 (251.0–463.0)	0.12
PIRCHE-II score for HLA class I, median (IQR)	243.5 (191.0–283.0)	155.5 (102.0–227.0)	0.22
PIRCHE-II score for HLA-A, median (IQR)	45.5 (21.0–95.0)	49.0 (26.0–103.0)	0.95
PIRCHE-II score for HLA-B, median (IQR)	82.0 (60.0 – 103.0)	39.5 (14.0–81.0)	0.08
PIRCHE-II score for HLA-C, median (IQR)	78.5 (58.0–110.0)	60.0 (34.0–79.0)	0.39
PIRCHE-II score for HLA class II, median (IQR)	267.0 (226.0–300.0)	203.5 (117.0–267.0)	0.13
PIRCHE-II score for HLA-DRB1, median (IQR)	53.5 (44.0–61.0)	40.0 (23.0–60.0)	0.28
PIRCHE-II score for HLA-DQA1/B1, median (IQR)	118.5 (96.0 – 136.0)	86 (49.0–143.0)	0.20
PIRCHE-II score for HLA-DPA1/B1, median (IQR)	26.0 (11.0–35.0)	9.5 (0.0–58.0)	0.75

*Statistically significant was lost after applied the Bonferroni correction for multiple testing (p<0.008).

AAMM, amino acid mismatches; IQR, interquartile range; IS, immunosuppression.

**Table 3:** Recipient HLA molecular mismatches in graft vs. host direction.

HLA mismatches	Patients On-IS (N=6)	Patients Off-IS (N=26)	Wilcoxon Exact Test p-value
*HLA Amino Acid mismatches (AAMM)*			
Total AAMM, median (IQR)	65.5 (60.0–114.0)	54.0 (34.0–68.0)	0.18
AAMM for HLA class I, median (IQR)	24.5 (19.0–38.0)	19.5 (9.0–30.0)	0.44
AAMM for HLA-A, median (IQR)	13.0 (0.0–17.0)	5.0 (0.0–8.0)	0.18
AAMM for HLA-B, median (IQR)	5.5 (5.0–9.0)	6.0 (3.0–12.0)	0.64
AAMM for HLA-C, median (IQR)	7.5 (2.0–10.0)	6.0 (3.0–9.0)	0.84
AAMM for HLA class II, median (IQR)	49.0 (26.0–84.0)	39.5 (19.0–45.0)	0.23
AAMM for HLA-DRB1, median (IQR)	14.0 (9.0–31.0)	13.0 (6.0–18.0)	0.43
AAMM for HLA-DQA_1_/B1, median (IQR)	38.5 (17.0–53.0)	18.0 (10.0–34.0)	0.12
*HLA EMS-3D score*			
Total EMS-3D score, median (IQR)	1.71 (1.32–2.70)	1.78 (1.78–1.22)	0.79
EMS-3D score for HLA class I, median (IQR)	1.11 (0.70–1.34)	1.10 (0.57–1.48)	0.84
EMS-3D score for HLA-A, median (IQR)	0.41 (0.0 – 0.66)	0.27 (0.0–0.48)	0.43
EMS-3D score for HLA-B, median (IQR)	0.27 (0.06–0.42)	0.13 (0.13–0.56)	0.53
EMS-3D score for HLA-C, median (IQR)	0.33 (0.20–0.54)	0.34 (0.31–0.62)	0.47
EMS-3D score for HLA class II, median (IQR)	0.65 (0.51 – 1.41)	0.70 (0.44–0.97)	0.56
EMS-3D score for HLA-DRB1, median (IQR)	0.25 (0.22–0.64)	0.30 (0.23–0.35)	0.90
EMS-3D score for HLA-DQA1/B1, median (IQR)	0.42 (0.29–0.77)	0.42 (0.21–0.55)	0.52
*Eplet mismatches*			
Total eplet mismatches, median (IQR)	88.5 (79.0–99.0)	92.0 (65.0–121.0)	0.88
HLA class I eplet mismatches, median (IQR)	30.5 (28.0–33.0)	34.0 (27.0–40.0)	0.55
HLA-A eplet mismatches, median (IQR)	14.0 (7.0–15.0)	13.0 (7.0–19.0)	0.97
HLA-B eplet mismatches, median (IQR)	9.0 (7.0–13.0)	11.0 (6.0–16.0)	0.50
HLA-C eplet mismatches, median (IQR)	7.0 (7.0–9.0)	10.0 (6.0–12.0)	0.75
HLA class II eplet mismatches, median (IQR)	60.0 (52.0–69.0)	64.5 (43.0–84.0)	0.79
HLA-DRB1 eplet mismatches, median (IQR)	42.0 (32.0–46.0)	42.5 (30.0–50.0)	0.80
HLA-DQA1/B1 eplet mismatches, median (IQR)	20.0 (17.0–23.0)	26.5 (13.0–33.0)	0.65
*PIRCHE-II score*			
Total PIRCHE-II score, median (IQR)	480.1 (455.0–508.0)	374.0 (238.0–536.0)	0.21
PIRCHE-II score for HLA class I, median (IQR)	204.0 (76.0–239.0)	148.0 (71.0–254.0)	0.88
PIRCHE-II score for HLA-A, median (IQR)	59.0 (0.0–105.0)	39.5 (0.0–69.0)	0.54
PIRCHE-II score for HLA-B, median (IQR)	34.0 (28.0–42.0)	63.0 (15.0–95.0)	0.53
PIRCHE-II score for HLA-C, median (IQR)	74.5 (6.0–108-0)	51.5 (15.0–110.0)	0.86
PIRCHE-II score for HLA class II, median (IQR)	304.5 (258.0–376.0)	195.0 (152.0–306.0)	0.14
PIRCHE-II score for HLA-DRB1, median (IQR)	46.5 (23.0–77.0)	37.0 (19.0–53.0)	0.58
PIRCHE-II score for HLA-DQA1/DQB1, median (IQR)	190.0 (73.0–216.0)	115.0 (68.0–188.0)	0.30
PIRCHE-II score for HLA-DPA1/DPB1, median (IQR)	0.0 (0.0–50.0)	0.0 (0.0–4.0)	0.51

AAMM, amino acid mismatches; IQR, interquartile range; IS, immunosuppression.

**Table 4** shows the individual PIRCHE-I score and antigen and allele HLA mismatches for all 32 recipients in both directions. Specifically for the two patients who developed GvHD (patients 21 and 25), PIRCHE-I scores were 0 and 8 in the GvH direction, respectively. Moreover, patient 21 with GvHD had the highest number (6) antigen and (6) allele HLA Class I mismatches in the GvH direction. However, five other patients who achieved high-level donor chimerism also had six HLA Class I allele mismatches and did not develop GvHD.

**Table 4:** Individual representation of PIRCHE-I score, antigen and allele HLA mismatches, and stem cell transplant outcome in both directionality (host versus graft [HvG] and graft versus host [GvH] direction).

Patient ID	Type of chimerism or no engraftment	PIRCHE-I score		HLA ABCDRDQ Antigen MM (10)				HLA ABCDRDQ Allele MM (12)			
				Class I (6)		DR/DQ (4)		Class I (6)		DRB1345/DQA1B1(6)	
		HvG	GvH	HvG	GvH	HvG	GvH	HvG	GvH	HvG	GvH
13	Mixed chimerism	15	5	2	2	1	2	2	2	3	3
18	Mixed chimerism	19	0	0	0	1	0	0	0	3	0
1	High-level donor chimerism	0	0	6	6	2	2	6	6	4	4
3	High-level donor chimerism	4	2	3	1	3	3	4	3	4	4
4	High-level donor chimerism	24	15	2	1	0	2	3	2	1	3
5	High-level donor chimerism	22	7	5	5	2	2	5	5	5	5
2	High-level donor chimerism	4	0	5	4	4	4	5	4	3	4
7	High-level donor chimerism	34	9	2	1	2	2	2	2	3	2
14	High-level donor chimerism	9	7	3	3	0	2	3	3	0	3
11	High-level donor chimerism	7	3	4	4	2	2	4	4	2	2
19	High-level donor chimerism	0	0	6	6	3	3	6	6	6	4
12	High-level donor chimerism	27	0	0	0	2	2	0	0	2	2
23	High-level donor chimerism	21	2	0	0	1	1	1	1	2	2
20	High-level donor chimerism	23	5	5	4	4	4	5	4	5	5
22	High-level donor chimerism	4	4	4	4	1	1	4	4	2	2
27	High-level donor chimerism	3	3	4	5	4	4	5	5	4	5
24	High-level donor chimerism	0	0	6	6	2	2	6	6	4	4
29	High-level donor chimerism	0	0	6	6	2	2	6	6	4	3
31	High-level donor chimerism	0	0	6	5	2	3	6	5	5	6
30	High-level donor chimerism	7	4	3	3	2	1	3	3	3	4
32	High-level donor chimerism	0	0	5	5	2	2	6	6	3	2
10	High-level donor chimerism	23	13	5	5	2	0	5	5	3	0
16	High-level donor chimerism	11	13	0	2	2	2	0	2	2	3
26	High-level donor chimerism	20	8	5	5	3	3	5	5	4	4
21	High-level donor chimerism with GvHD	0	0	6	6	1	1	6	6	2	2
25	High-level donor chimerism with GvHD	20	8	4	4	3	3	5	5	6	5
8	Transient chimerism	26	11	2	3	2	2	4	4	3	3
9	Transient chimerism	7	1	5	5	2	3	5	5	4	6
17	Transient chimerism	24	0	2	0	2	2	2	0	3	3
15	Transient chimerism	19	10	5	4	2	2	5	4	3	3
6	No engraftment	0	0	6	6	1	3	6	6	5	5
28	No engraftment	0	0	4	5	4	2	4	5	5	3

Colored bars represent the outcome of the stem cell transplant (light green—high-level donor chimerism, darker green—mixed chimerism; blue—transient chimerism, and gray–no engraftment). Any HLA mismatch (number >0) is shown in bold and the highest number of mismatches per category is highlighted in red.

MM, mismatches; HvG, host versus graft; GvH, graft versus host; GvHD, graft versus host disease.

### Non-permissiveness of DPB1 T-cell epitopes and stem cell transplant outcome

We next sought to determine whether HLA-DPB1 mismatches could be an indicator for different outcomes post-transplant. Based on T-cell-epitope groups (TCE-Groups), it has been demonstrated that HLA-DPB1 mismatches can be divided into two categories: permissive, mismatches that may be well tolerated, and non-permissive, mismatches with an increased risk for adverse effects after hematopoietic stem cell transplantation. Two transplant pairs did not have enough DNA to perform DPB1 typing (patient 22 and 23). For the rest of the cohort, we classified the HLA-DPB1 mismatches based on TCE-Groups (**Table 5**). Seven of the patients had high and/or intermediate non-permissive mismatched DPB1 T-cell epitopes; however, five out of seven (71%) still achieved high-level donor chimerism. Interestingly, both patients who developed GvHD had permissive HLA-DPB1 mismatches.

**Table 5:** Non-permissiveness of DPB1 T-cell epitope mismatch, HLA-B leader sequence mismatch, and KIR ligands mismatch analysis (missing self).

Patient ID	Type of chimerism or no engraftment	DPB1 Non-Permissive T-cell-epitope MM		HLA-B Leader Mismatch	KIR ligands MM	
		HvG	GvH		HvG	GvH
13	Mixed chimerism	No	No	No	**1**	**1**
18	Mixed chimerism	No	No	No	0	0
1	High-level donor chimerism	No	No	**Yes**	0	**1**
3	High-level donor chimerism	No	No	**Yes**	0	**1**
4	High-level donor chimerism	No	No	**Yes**	0	0
5	High-level donor chimerism	No	No	**Yes**	0	0
2	High-level donor chimerism	No	No	**Yes**	**1**	**1**
7	High-level donor chimerism	No	No	No	0	**1**
14	High-level donor chimerism	No	No	**Yes**	0	0
11	High-level donor chimerism	No	No	No	0	**1**
19	High-level donor chimerism	No	No	**Yes**	**1**	0
12	High-level donor chimerism	No	No	No	0	0
23	High-level donor chimerism	/	/	No	0	0
20	High-level donor chimerism	No	No	No	**1**	0
22	High-level donor chimerism	/	/	No	0	0
27	High-level donor chimerism	**High**	**Intermediate**	**Yes**	**1**	**1**
24	High-level donor chimerism	No	No	**Yes**	**2**	0
29	High-level donor chimerism	No	No	**Yes**	**1**	0
31	High-level donor chimerism	No	**High and Intermediate**	**Yes**	**2**	0
30	High-level donor chimerism	No	No	No	0	**1**
32	High-level donor chimerism	**High and Intermediate**	No	No	**1**	0
10	High-level donor chimerism	No	**Intermediate**	**Yes**	0	0
16	High-level donor chimerism	No	No	**Yes**	**1**	0
26	High-level donor chimerism	**Intermediate**	No	**Yes**	0	**1**
21	High-level donor chimerism with GvHD	No	No	**Yes**	0	**1**
25	High-level donor chimerism with GvHD	No	No	**Yes**	**1**	0
8	Transient chimerism	No	No	**Yes**	**1**	0
9	Transient chimerism	**Intermediate**	No	No	0	0
17	Transient chimerism	No	No	**Yes**	0	**2**
15	Transient chimerism	No	No	No	0	0
6	No engraftment	No	No	**Yes**	**1**	0
28	No engraftment	No	**Intermediate**	**Yes**	0	**2**

MM, mismatches; HvG, host versus graft; GvH, graft versus host; GvHD, graft versus host disease.

Colored bars represent the outcome of the stem cell transplant (light green—high-level donor chimerism; darker green—mixed chimerism; blue—transient chimerism; and gray—no engraftment). Any mismatch is shown in bold and the highest mismatch per category is highlighted in red.

### HLA-B leader mismatch and outcome after combined kidney and stem-cell transplant

The HLA-B leader sequence at position −21 encodes methionine (M) or threonine (T) and can give rise to three TT, MT, or MM genotypes, and it has been shown that patients with HLA-B leader sequence mismatched donors have a higher risk of GvHD when the patient has an M in the leader sequence (17). Using the online tool, we calculated the HLA-B types for each donor and recipient and compared them for mismatches in the B leader sequences (**Table 5**). While both patients with high-level donor chimerism and GvHD were mismatched in the HLA-B leader sequence, 14 other patients with high-level donor chimerism were also mismatched and did not develop GvHD. In addition, half of the transplant pairs with HLA-B leader mismatches had the GvHD risk M type in the recipient, but only one developed GvHD.

### Mismatches between KIR ligands and predicted NK cell alloreactivity in combined kidney and stem-cell transplant setting

Finally, to predict natural killer (NK) cell alloreactivity, we used the ligand calculator on the IPD-IMGT/HLA website and determined which KIR ligands were present based on the HLA typing of the patient and donor and calculated the KIR ligand mismatches (missing-self) for each transplant pair (**Table 5**). In total, 22/32 patients had KIR ligand mismatches, of which four had two mismatches. Patients with GvHD had one KIR ligand mismatch but in the opposite direction, one in GvH and the other in the HvG direction.

## Discussion

In the current study, we performed high-resolution HLA typing and investigated the association of HLA molecular mismatching in the induction of donor-specific acquired tolerance after combined kidney/hematopoietic stem cell transplantation and achievement of stable donor chimerism. To our knowledge, this is the first study to investigate the possible role of HLA molecular mismatches in this transplant setting to date. Our results showed a trend for significance between HLA-DRB1 molecular mismatch scores at the HvG direction and donor-specific stable chimerism after combined kidney and FCR transplant. Of all the different HLA mismatch modalities, only the EMS-3D score for HLA-DRB1 molecules was initially statistically associated with achieving donor-specific stable chimerism; however, this significance was lost after applying the Bonferroni correction. Finally, the non-permissiveness of HLA-DPB1 T-cell epitope group mismatches, HLA-B leader sequence mismatches, and KIR ligands mismatches did not predict the studied clinical outcomes in this HLA-mismatched and combined kidney/FCR transplant patient cohort.

Using all available HLA molecular mismatch modalities, this study found that HLA class II, particularly HLA-DRB1, molecular mismatches in HvG direction, may be more relevant than HLA class I molecular mismatches in achieving donor-specific stable chimerism after combined stem cell and kidney transplantation. This finding supports the recent literature in kidney transplantation that suggests greater clinical importance of HLA class II molecules, mainly HLA-DR and HLA-DQ, than HLA class I molecules (9–12, 18). In contrast, a recent study performed in a haploidentical hematopoietic stem cell transplant cohort found that the number of HLA-mismatched eplets in the HvG direction was not associated with any major clinical outcomes such as overall survival, disease-free survival, non-relapse mortality, relapse, and GvHD (19). However, they discovered a strong correlation between total HLA class II eplet mismatches in the GvH direction and slower neutrophil and platelet engraftment, likely due to eplet mismatches derived from HLA-DRB1 molecules. Interestingly, their analysis by quartiles revealed that the significant findings were driven mainly by the lowest quartile, which includes matched pairs, compared to the upper three quartiles without any dose effect. This further suggests that delayed engraftment is likely driven by some MM with particular characteristics (immunogenic) rather than the total number of all mismatched eplets. Our study’s finding that only the EMS-3D score showed the strongest association with achievement of donor-specific stable chimerism supports this and suggests that the electrostatic disparity at the tertiary level of certain HLA mismatches is likely important and relevant to their immunogenicity (16).

Although the development of *de novo* DSA was a scarce event in this cohort, the three DQ *dn*DSAs were still informative. Two DQ DSAs developed against high eplet mismatch loads (13 and 14 eplet MM), while one dnDSA was formed against the donor’s DQ7 molecule, which had only two mismatched eplets. This finding contradicts some studies that proposed safe eplet mismatch load thresholds for *de novo* DSA occurrence and risk stratification (11), supporting that there is no universal safe threshold for eplet mismatch load.

The effect of T-cell-epitope matching at HLA-DPB1 locus in recipients of unrelated-donor hemopoietic-cell transplantation has been well established (20). The classification of HLA-DPB1 mismatches based on T-cell-epitope groups (TCE-Groups) helps determine mismatches that may be tolerated (permissive) and those that increase the risk (non-permissive) of acute GvHD. However, in our combined kidney/FCR transplant patients, matching at the HLA-DPB1 locus did not seem to have the same effect. In fact, 71% of patients with non-permissive DPB1 mismatches still achieved high-level donor chimerism, while two patients who developed GvHD had permissive HLA-DPB1 mismatches. This likely suggests that DPB1 non-permissive mismatches may not be as relevant when other, more immunogenic HLA loci are mismatched.

Finally, we interrogated the possible impact of KIR ligand mismatches and HLA-B leader sequence mismatch and found no correlation with clinical outcomes in this cohort of induced acquired tolerance. These results contribute to the ongoing discussion about the clinical relevance of KIR ligand mismatches and natural killer (NK) cell alloreactivity in allogeneic stem cell transplantation (21). However, our findings contrast those of a previous study by Petersdorf et al. in 2020, which showed that patients with HLA-B leader sequence mismatched donors were at a higher risk of GvHD when the patient had an M in the leader sequence (17). Of the 20 HLA-B leader sequence mismatched transplant pairs in our cohort, only two patients developed GvHD, and only one had the risk M type. The same explanation could be valid for B leader MM that other more immunogenic mismatches might be more clinically relevant in this transplant setting.

Our study has some limitations. This is a single-center study with mainly a male adult subject population; therefore, our results might not be generalizable to other populations. Additionally, our study may not have been adequately powered to detect all differences in HLA molecular mismatches for all HLA loci between the two groups. For instance, the clinical relevance of the molecular mismatches for the HLA-DQ molecule, which is well documented, might not have been detected in our study due to the small sample size (9–12). In addition, we used the current algorithm for calculating eplet mismatches, which relies on an incomplete list of mainly theoretically predicted eplets. Finally, although we assessed the KIR ligand mismatch, we did not perform KIR receptor genotyping of the recipients. Further work is needed to validate our findings in a larger cohort.

In conclusion, our results suggest only a possible association of HLA-DRB1 molecular mismatches and achieving stable chimerism, mostly when the electrostatic quality of the mismatch is considered. Directionality of non-permissive HLA-DPB1 T-cell-epitope group mismatches, HLA-B leader sequence mismatch, and directionality of KIR ligands mismatches did not predict the clinical outcome in this combined transplant patient cohort. New tools/approaches to assess immunogenicity for the mismatched HLA alleles beyond counting the number of HLA molecular mismatches should be considered for optimal risk stratification and candidate selection.

## Data availability statement

The raw data supporting the conclusions of this article will be made available by the authors, without undue reservation.

## Ethics statement

The studies involving humans were approved by the Northwestern University Institutional Review Board and the FDA (IDE 13947). The studies were conducted in accordance with the local legislation and institutional requirements. The participants provided their written informed consent to participate in this study.

## Author contributions

AS: Conceptualization, Formal analysis, Methodology, Visualization, Writing – original draft. AT: Conceptualization, Data curation, Resources, Writing – review & editing, Supervision. VK: Writing – review & editing, Methodology. HC: Writing – review & editing, Methodology. CS: Data curation, Writing – review & editing. CU: Writing – review & editing, Data curation. SI: Writing – review & editing, Methodology. JL: Data curation, Investigation, Resources, Supervision, Writing – review & editing.
